# 

**Published:** 1904-02-13

**Authors:** 


Crown 8vo. Illustrated. Price **2s. 6d.** net.

**ANÆSTHETICS.** By J. Blumfeld, M.D.Cantab., Senior Anæsthetist to St. George's Hospital; Anæsthetist to the Grosvenor Hospital for Women and Children, London, and to the National Dental and Metropolitan Hospitals.

*Lancet.*—"....In writing this handbook the author has been quite successful." *Australasian Medical Gazette.*—"We can strongly commend this book." *Medical Chronicle.*—"The book is one which we can heartily recommend to senior students and practitioners."

London: Baillière, Tindall & Cox, 8 Henrietta St., Covent Garden.

---

Second Edition. Crown 8vo., price **3/6** net.
With original "Plea—1900—for the State Registration of Nurses."

**MOCK NURSES OF THE LATEST FASHION.**

By FREDERICK J. GANT, F.R.C.S.,
Consulting Surgeon to the Royal Free Hospital.

BAILLIÈRE, TINDALL & COX,
Henrietta Street, Covent Garden, London.

---

**COTTAGE HOSPITALS, GENERAL, FEVER AND CONVALESCENT.**

Their Progress, Management and Work in Great Britain, Ireland and the United States of America.

By Sir HENRY BURDETT, K.C.B.

Third Edition, profusely Illustrated, with nearly 50 Plans, Diagrams, &c. Crown 8vo. cloth gilt, **10/6** post free.

"A complete manual of all that relates to the founding, the cost and the management of cottage hospitals. No more complete manual could be required."—*St. James's Gazette.*

*Of all Booksellers.*

Published by THE SCIENTIFIC PRESS, LIMITED,
28 & 29 Southampton St., Strand, London, W.C.

### NOTICE TO CORRESPONDENTS.

All MSS., Letters, Books for Review, and other matters intended for the Editor should be addressed THE EDITOR, "THE HOSPITAL." THE HOSPITAL BUILDINGS, 28 & 29 SOUTHAMPTON STREET, STRAND, W.C.

The Editor cannot undertake to return rejected MS., even when accompanied by stamped directed envelope.

**Notice to Advertisers and Subscribers.**

All Advertisements, Orders for Copies of THE HOSPITAL, and Business Communications should be addressed to **THE MANAGER (Not to the Editor),** THE HOSPITAL, 28 & 29 Southampton Street, Strand, London, W.C.

The Cost of Subscription, post free, is as follows:—Per week, 3½d.; per quarter, 3s. 9d.; per half-year, 7s. 6d.; per annum, 15s. Foreign and Colonial:—Per annum, 19s. 6d., payable, in all cases, in advance.

**NOTICE.—Vol. XXXIV. of "The Hospital" (April 1903 to September 1903), in handsome cloth binding, is now on sale at the office of this Journal, price 10s. 6d. Cases for binding the Numbers contained in this Volume 2s. Index to Vol. XXXIV., 2½d., post free.**

**The Monthly part for February is now ready. Price 1s., by post, 1s. 3d.**

### BOOKS RECEIVED.

A. and C. Black.

"Who's Who, 1904."

"Who's Who Year Book, 1904."

"The Englishwoman's Year Book, 1904."

H. J. Glaisher.

"Charles White, F.R.S." A great Provincial Surgeon and Obstetrician of the Eighteenth Century. By C. J. Cullingworth, M.D.

Zoological Society.

"Protozoa." By H. Woodward.

### The Student of Medicine.

### APPOINTMENTS.

**W. Victor Bonney**, M.S., M.D.Lond., F.R.C.S., M.R.C.S., Lecturer in Practical Midwifery in the Middlesex Hospital Medical School. **Edred M. Corner**, M.B., B.C.Cantab., F.R.C.S., Assistant Surgeon to St. Thomas's Hospital. **Sidney R. Dyer**, M.D.Brux., L.R.C.P., M.R.C.S., Principal Medical Officer of Dartmouth Convict Establishment. **H. L. Eason**, M.D., M.S.Lond., Dean of Guy's Hospital Medical School. **W. D. Harmer**, M.C.Cantab., F.R.C.S.Eng., Surgeon to the Metropolitan Hospital, Kingsland Road. **John Hay**, M.D., M.R.C P., Physician to the Liverpool Stanley Hospital. **W. E. Heilborn**, M.B., B.C.Cantab., Honorary Assistant Medical Officer to the Bradford Children's Hospital. **Percy Holgate**, M.B., B.S.Durh., Medical Officer to the Tynemouth Township of the Tynemouth Union. **L. E. Jowers**, F.R.C.S.Edin., L.R.C.P.Lond., Assistant Surgeon to the Hastings, St. Leonards, and East Sussex Hospital. **Percy R. Mander**, M.D.Durh., M.R.C.S.Eng., Medical Officer to H.M. Prison, Stafford. **Norman G. Meade**, M.R.C.S., L.R.C.P.Lond., Honorary Assistant Medical Officer to the Bradford Children's Hospital. **W. Pattullo**, L.R.C.P. and S.Edin., L.F.P.S.Glasg., District Medical Officer of the Durham Union. **James Phillips**, F.R.C.S.Edin., L.R.C.P.Lond., Honorary Medical Officer to the Bradford Children's Hospital. **W. George Porter**, M.D., M.R.C.S., F.R.C.P., Clinical Assistant to the Chelsea Hospital for Women. **J. Dallas Pratt**, M.D.Dub., B.Ch., F.R.C.S.I., Certifying Factory Surgeon for the Dublin District. **R. Wynne Rouw**, M.R.C.S., L.D.S., L.R.C.P.Lond., Dental Surgeon and Lecturer on Dental Surgery at Guy's Hospital. **Joseph Hambley Rowe**, M.B., C.M., Anæsthetist to the Bradford Children's Hospital. **Sydney Scott**, B.S.Lond., F.R.C.S.Eng., Honorary Surgeon to the Westminster General Dispensary, London. **William Robert Smith**, M.D., B.S.Lond., F.R.C.S. Eng., Assistant Surgeon to the Hospital for Women, Nottingham. **H. J. Waring**, M.S.Lond., F.R.C.S.Eng., Consulting Surgeon to the Metropolitan Hospital, Kingsland Road. **C. Gordon Watson**, F.R.C.S.Eng., Assistant Surgeon to the Metropolitan Hospital, Kingsland Road. **E. Lloyd Williams**, M.B., C.M.Edin., Medical Officer and Public Vaccinator for the Llanrwst District and Workhouse of the Llanrwst Union.

### "The Hospital" Institutional Library.

**"Hospitals and Asylums of the World." 4 Vols., with a Portfolio of Plans, complete £12 12s.**

**"Burdett's Hospitals and Charities, 1903." 5s. net.**

**"Burdett's Official Nursing Directory, 1903." 3s. net.**

**"Cottage Hospitals, General, Fever, and Convalescent." 10s. 6d.**

**"Uniform System of Accounts for Hospitals and Public Institutions." New Edition. 4s. net.**

**"Hospital Expenditure: The Commissariat." 2s. 6d.**

**"A Legal Handbook for the Use of Hospital Authorities." 2s. 6d.**

**"The Cottage Hospital Case Book or Register of Patients." 10s. and 12s. 6d.**

**All these are published by the Scientific Press, Ltd., and may be obtained through any bookseller, or direct from the publishers, 28 and 29 Southampton Street, Strand, London, W.C.**

### How to Become a Nurse.

### THE NURSING PROFESSION: HOW AND WHERE TO TRAIN.

2/4 Post free.

By SIR HENRY BURDETT, K.C.B.

Is a reliable and up-to-date Guide to training for the profession of a Nurse, and contains all the information a would-be nurse requires.

### NURSING TEXT-BOOKS.

Post free.

3/6 **NURSING: ITS THEORY AND PRACTICE.**
A Complete Text-Book of Medical, Surgical, and Monthly Nursing.
By PERCY G. LEWIS, M.D., M.R.C.S., L.S.A.
New and Enlarged E lition. Over 400 pp. profusely illustrated.

2/6 **ELEMENTARY ANATOMY AND SURGERY FOR NURSES.**
By WILLIAM McADAM ECCLES, M.S.Lond., M.B., &c.
Illustrated, bound in cloth.

2/- **ELEMENTARY PHYSIOLOGY FOR NURSES.**
By C. F. MARSHALL, M.D., B.Sc., F.R.C.S.
Illustrated, bound in cloth.

2/- **PRACTICAL GUIDE TO SURGICAL BANDAGING AND DRESSINGS.**
By WILLIAM JOHNSON SMITH, F.R.C.S.
Suitable size for apron pocket, profusely illustrated, bound in cloth.

2/- **THE NURSE'S DICTIONARY OF MEDICAL TERMS AND NURSING TREATMENT.**
Compiled for the use of Nurses. By HONNOR MORTEN.
Suitable for the apron pocket, bound in cloth boards, 160 pp.

1/1 **THE EXAMINATION OF THE URINE.**
By J. K. WATSON, M.D., M.B., C.M., Author of "A Handbook for Nurses."
Demy 16mo. cloth boards.

*These books are obtainable of all Booksellers and are published by*
THE SCIENTIFIC PRESS, LTD., 28 and 29 Southampton Street, Strand, London, W.C.,
*who will send their latest Catalogue of Nursing Manuals, post free, to any Nurse on application.*

